# Transcriptional Profiling and miRNA-Target Network Analysis Identify Potential Biomarkers for Efficacy Evaluation of Fuzheng-Huayu Formula-Treated Hepatitis B Caused Liver Cirrhosis

**DOI:** 10.3390/ijms17060883

**Published:** 2016-06-03

**Authors:** Qilong Chen, Feizhen Wu, Mei Wang, Shu Dong, Yamin Liu, Yiyu Lu, Yanan Song, Qianmei Zhou, Ping Liu, Yunquan Luo, Shibing Su

**Affiliations:** 1 Research Center for Traditional Chinese Medicine Complexity System, Shanghai University of Traditional Chinese Medicine, Shanghai 201203, China; cqlw1975@126.com (Q.C.); wenli228@163.com (S.D.); cqlw0218@163.com (Ya.L.); ava0048@163.com (Yi.L.); ynsong@163.com (Y.S.); tazhou@163.com (Q.Z.); 2 Laboratory of Epigenetics, Institute of Biomedical Science, Fudan University, Shanghai 200032, China; qyp2015@126.com; 3 Department of Endocrinology and Metabolism, Shanghai First People’s Hospital, Shanghai Jiaotong University, Shanghai 201201, China; cql0218@163.com; 4 Institute of Liver Disease, Shanghai University of Traditional Chinese Medicine, Shanghai 201203, China; liupingg1@126.com; 5 Shuguang Hospital Affiliated to Shanghai University of Traditional Chinese Medicine, Shanghai 201203, China

**Keywords:** Fuzheng-Huayu (FZHY) formula, hepatitis B-caused cirrhosis (HBC), microRNA, miRNA-target network

## Abstract

Fuzheng-Huayu (FZHY) formula has been found to have a satisfactory effect on hepatitis B-caused cirrhosis (HBC) treatment. However, the efficacy evaluation of FZHY is often challenging. In this study, a randomized, double-blind and placebo-controlled trial was used to evaluate the therapeutic efficacy of FZHY in HBC treatment. In the trial, 35 medical indexes were detected, and 14 indexes had a statistically-significant difference before compared to after the trial. Importantly, the Child-Pugh score also demonstrated FZHY having therapeutic efficacy. Furthermore, the microRNA (miRNA) profiles of 12 serum samples were detected in FZHY groups, and 112 differential-expressed (DE) miRNAs were determined. Using predicted miRNA targets, 13 kernel miRNAs were identified from the established miRNA-target network. Subsequently, quantitative Real-time Polymerase Chain Reaction (qRT-PCR) was used to validate the expression level of 13 identified miRNAs in the trials. The results showed that nine miRNAs have a statistically-significant difference before compared to after FZHY treatment. By means of a logistic regression model, a miRNA panel with hsa-miR-18a-5p, -326, -1182 and -193b-5p was established, and it can clearly improve the accuracy of the efficacy evaluation of FZHY. This study suggested that the particular miRNAs can act as potential biomarkers and obviously increase the diagnostic accuracy for drug evaluation in HBC treatment progression.

## 1. Introduction

Liver cirrhosis is a common pathological consequence of chronic liver disease, which is characterized by liver fibrosis, scar tissue and regenerative nodules, as well as leading to the destruction of the hepatic microstructure and liver dysfunction. Hepatitis B virus (HBV) is one of the most common etiologies of liver cirrhosis in China, and annually, about 1.5 million people worldwide suffer from hepatitis B-caused cirrhosis (HBC) [1]. The five-year survival rate of patients with severe HBC is only about 50% [2], and hepatocellular carcinoma (HCC), especially, was estimated to occur almost exclusively in patients with HBC [3]. In the past few decades, there has been a lack of effective clinical drugs for the therapy of HBC; fortunately, a Chinese herbal recipe, Fuzheng-Huayu (FZHY) formula, is a prospect for HBC treatment.

The Fuzheng-Huayu (FZHY) formula is one of the most well-studied anti-fibrotic products [4] and is approved by the Chinese State Food and Drug Administration (SFDA) in China. It contains six Chinese medicinal herbs [5], such as *Radix Salvia Miltiorrhizae* (Dan-shen), *Cordyceps* (Chong-cao), *Semen Persicae* (Tao-ren), *Gynostemma Pentaphyllammak* (Jiao-gulan), *Pollen Pini* (Song-huafen) and *Fructus Schisandrae Chinensis* (Wu-weizi), formulated based on Chinese medicine theory of liver fibrosis treatment [6]. Its efficacy against liver fibrosis was confirmed in a phase II clinical trial carried out in the U.S. Pharmacological and clinical studies demonstrated that FZHY can work against liver fibrosis, especially in treating liver fibrosis and cirrhosis caused by chronic hepatitis B (CHB) [7,8,9]. FZHY might suppress hepatocyte apoptosis to inhibit liver fibrosis by regulating mediators in death receptor and mitochondrial pathways [10]. Importantly, FZHY can regulate the transforming growth factor-β1 (TGF-β1) signaling transduction pathway [11,12,13], which was considered as a pivotal pathway against liver fibrosis progression.

Although the FZHY formula is advantageous for HBC treatment, the efficacy evaluation of FZHY is often challenging. To this day, several serum markers have been well evaluated in diagnosing liver cirrhosis, including FibroTest, Aspartate aminotransferase-to-Platelet Ratio Index (APRI), the prothrombin index (PI), aspartate aminotransferase (AST) to alanine aminotransferase (ALT) ratio (AAR), the Lok index and the Goteborg University cirrhosis index (GUCI) [14,15]. However, although marker levels are highly reproducible, they are not specific for liver disease [16] and do not allow easy discrimination of the intermediate stages of HBC in treatment progression. It is suggested that there is difficulty in distinguishing it from medical indexes’ criteria alone. If the efficacy evaluation of FZHY could be diagnosed in a timely manner, the clinical utility of biomarkers in the diagnosis of the intermediate stages of HBC treatment would require further verification.

MicroRNAs (miRNAs) are a class of approximately 22 nucleotides, endogenous, non-coding and single-stranded small RNA. At the translational level, miRNA can guide the degradation of the target mRNA silencing complex (RISC) and negatively regulate the expression of target mRNA [17,18,19]. In eukaryotic cells, miRNAs can regulate more than 30% of the target genes and partake in multiple regulatory pathways, such as cell proliferation, differentiation, development, aging and apoptosis. This suggests that miRNA might act as potential biomarkers for the diagnosis of disease, as well as prognosis.

As a paradigm of the FZHY formula curative estimation, here, we report that the medical index (MI) levels were prominently changed in FZHY-treated HBC patients; especially, the Child–Pugh scores were statistically significantly different before compared to after FZHY treatment. These data showed that FZHY formula has dramatic therapeutic efficacy in HBC treatment, although it was only evaluated from clinical data. Furthermore, we hypothesize that miRNAs’ expression levels are ideal biomarkers to evaluate the therapeutic efficacy of FZHY. Using miRNA profiles, we functionally characterize the significant impact of 13 kernel miRNAs by an established miRNA-network. Based on quantitative Real-time Polymerase Chain Reaction (qRT-PCR) and the logistic regression model, a miRNA panel with four miRNAs was identified, which improves the diagnostic accuracy of the efficacy evaluation of FZHY. These results contribute to our current understanding of the characteristics of FZHY curative estimation in HBC treatment progression.

## 2. Results

### 2.1. The Synopsis of Fuzheng-Huayu (FZHY)-Treated Hepatitis B-Caused Cirrhosis (HBC) Patients

**Figure 1:**
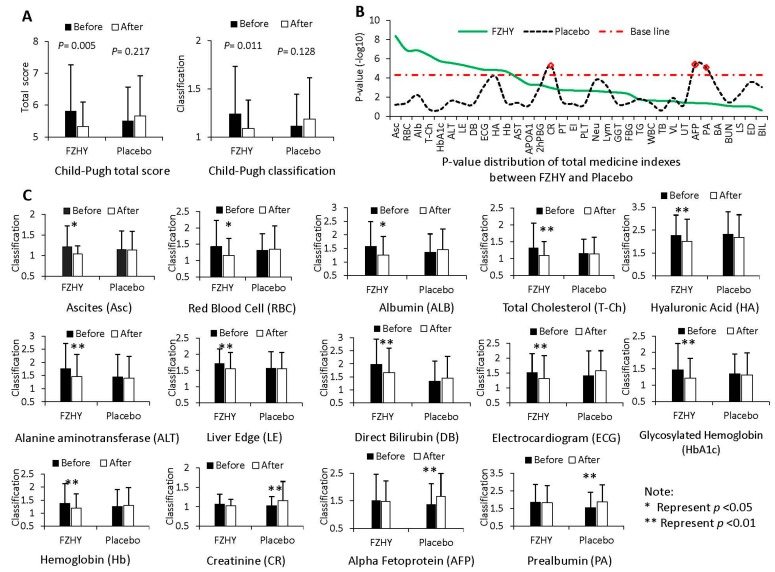
The clinical data analysis results between before and after treatment of Fuzheng-Huayu (FZHY) and placebo groups. (**A**) The Child–Pugh total score and classification comparison between FZHY and placebo groups; (**B**) the *p*-values’ distribution of medical indexes, which were tested using the random variance model of R package; (**C**) the expression levels of 14 significant medical indexes between FZHY and the placebo groups. To draw the figures conveniently, the detected value of each medical index was assigned a code (Arabic numerals); for example, 1 represents that the detected value is the normal level; 2 represents that the value is the abnormal level, but not worse; and 3 represents that the detected value is the abnormal level and worse.

After six months of treatment, the Child-Pugh total score and classification have significant changes (Child-Pugh total score, *p* = 0.005; Child-Pugh classification, *p* = 0.011) before compared to after FZHY treatment; however, as a comparison, these parameters do not have statistically significant differences in the placebo group (Child-Pugh total score, *p* = 0.217; Child-Pugh classification, *p* = 0.128; Figure 1A). The results suggest that the therapeutic efficacies of FZHY and placebo are different in the clinical trial, and FZHY treatment is able to improve the Child-Pugh score of HBC patients obviously.

Furthermore, the *p*-value distribution of 35 MIs is shown in Figure 1B, and 14 MIs were identified to be significant between the FZHY and placebo groups. In these 14 identified MIs, 11 MIs were obtained from the FZHY group, and three MIs were selected from the placebo group (Figure 1C). This suggests that FZHY can regulat these medical indexes’ expression levels after treatment in the clinic, at least in part; it might have an inhibiting effect for several indexes such as Creatinine (CR), Alpha Fetoprotein (AFP) and Prealbumin (PA) in HBC treatment progression. Interestingly, we notice that some of the identified MIs have no biological relevance (Red Blood Cell and Hemoglobin, for example) between before and after therapy with FZHY, because this random association could be detected in a large enough dataset. However, in fact, the random association of these identified MIs also can provide useful ancillary functions to demonstrate the response to FZHY in HBC treatment process.

### 2.2. Differentially-Expressed miRNAs and Target Genes’ Enrichment Analysis

**Figure 2:**
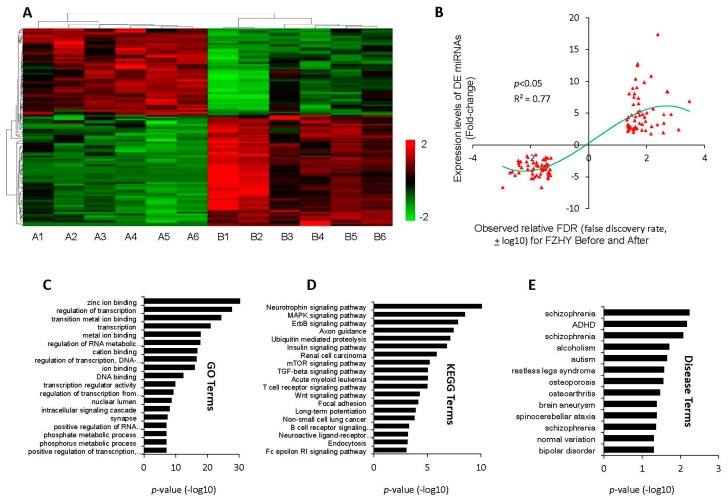
The bioinformatics analysis of miRNAs and target genes between before and after FZHY treatment for hepatitis B-caused cirrhosis (HBC) patients. (**A**) Heat-map of the differential expression of miRNAs between before and after FZHY treatment. Red color represents that the miRNA is upregulated, and green color represents downregulated. The relationship among the samples was divided by binary tree classification and is shown in the upper portion. The hierarchical cluster of miRNAs is displayed at the bottom; (**B**) The distribution of differential expressed (DE) miRNA expression levels; the right side represents the up-expressed miRNAs between before and after FZHY treatment; the left side represents the down-expressed miRNAs between before and after FZHY treatment; (**C**–**E**) MiRNA target genes related Gene ontology (GO) terms, Kyoto Encyclopedia of Genes and Genomes (KEGG) terms and disease terms.

In this work, 112 differentially-expressed (DE) miRNAs were identified between before and after FZHY-treated HBC patients, including 63 upregulated miRNAs and 49 downregulated miRNAs (Figure 2A,B). Using the prediction programs, the miRNA target genes were predicted. After redundancy analysis, the final database of 112 DE miRNAs with 5161 target genes was built.

To understand these miRNAs holistically, we conducted functional enrichment analysis for the target genes using DAVID (The Database for Annotation, Visualization and Integrated Discovery) online analysis [20,21]. GO (Gene ontology) term (10% top terms) analysis reveals that the miRNA-targets are mainly associated with metal ion binding, transition metal ion binding, regulation of transcription, regulation of the RNA metabolic process, DNA binding, transcription regulator activity, regulation of transcription from the RNA polymerase II promoter, positive regulation of the RNA metabolic process, positive regulation of transcription, phosphate and the phosphorus metabolic process (Figure 2C). KEGG (Kyoto Encyclopedia of Genes and Genomes) pathways show an impressive functional association with various signal-related pathways, such as the neurotrophin signaling pathway (*p* = 5.45 × 10^−11^), the MAPK signaling pathway (*p* = 3.02 × 10^−9^), the ErbB signaling pathway (*p* = 1.37 × 10^−8^), the insulin signaling pathway (*p* = 1.44 × 10^−7^), the mTOR signaling pathway (*p* = 5.98 × 10^−6^), the TGF-β signaling pathway (*p* = 9.16 × 10^−6^), the T cell receptor signaling pathway (*p* = 9.77 × 10^−6^), the Wnt signaling pathway (*p* = 5.26 × 10^−5^), as well as the B cell receptor signaling pathway (*p* = 5.34 × 10^−4^) and the Fc epsilon RI signaling pathway (*p* = 9.20 × 10^−4^) (Figure 2D). Furthermore, axon guidance, ubiquitin-mediated proteolysis, renal cell carcinoma, acute myeloid leukemia, non-small cell lung cancer, focal adhesion, long-term potentiation, neuroactive ligand-receptor interaction and endocytosis also have a high correlation with these target genes.

Furthermore, the disease terms show that these target genes are mainly associated with developmental, neurological and psychological diseases, such as schizophrenia, attention-deficit hyperactivity disorder (ADHD), alcoholism, autism, osteoporosis, brain aneurysm, spinocerebellar disease, normal variation and bipolar disorder (Figure 2E). Although none of the disease terms appear relevant for hepatitis B or fibrosis, however, these terms in fact are related to the complications and often occur with liver cirrhosis. This result suggests that FZHY can obviously improve the quality of life of patients.

### 2.3. miRNA-Target Network Building and Potential miRNA Marker Screening

**Figure 3:**
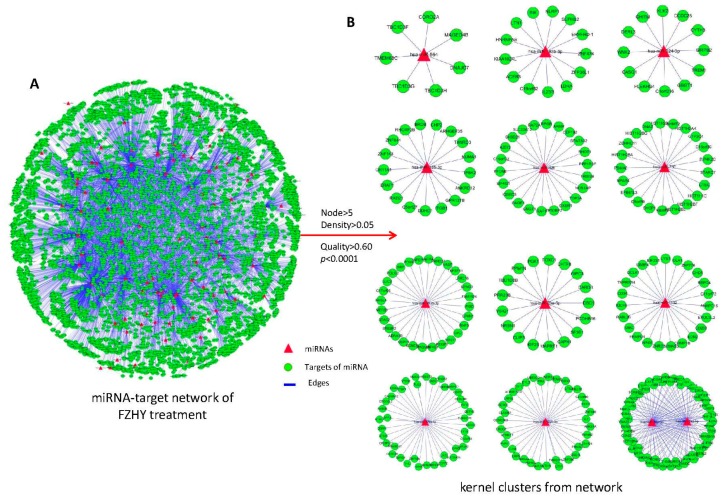
The miRNA-target network was constructed based on DE miRNAs and target genes, and the related clusters were identified using the ClusterONE algorithm. (**A**) The global profiles of the FZHY treatment-related miRNA-target network were built. The target genes of miRNAs were predicted using the TarBase (v7.0), miRecords and miRTarBase databases and the miRanda, miRDB, miRWalk and RNAhybrid prediction programs; (**B**) The kernel miRNA-related clusters isolated from the network using the ClusterONE algorithm, which was defined as node > 5, density > 0.05, quality > 0.60 and *p* < 0.0001. There are 12 clusters, containing 13 kernel miRNAs.

Using the DE miRNAs and target genes, the miRNA-target network was constructed. As shown in Figure 3A, the topological profile of the network is more likely similar to the “Medusa” model [22], which consists of a regulatory core structure by kernel nodes and represents most prominently the functions in the network. This suggests that the kernel nodes are determinants in the realized network profiles, while the periphery nodes only should be regulated [23]. For this reason, 12 clusters with 13 kernel miRNAs were isolated from the miRNA-target network based on the ClusterONE algorithm [24] (Figure 3B, Table 1). These kernel miRNAs might play important roles in HBC treatment progression and also might act as potential biomarkers for the efficacy evaluation of FZHY.

### 2.4. Establishing and Validating the Potential miRNA Markers

**Figure 4:**
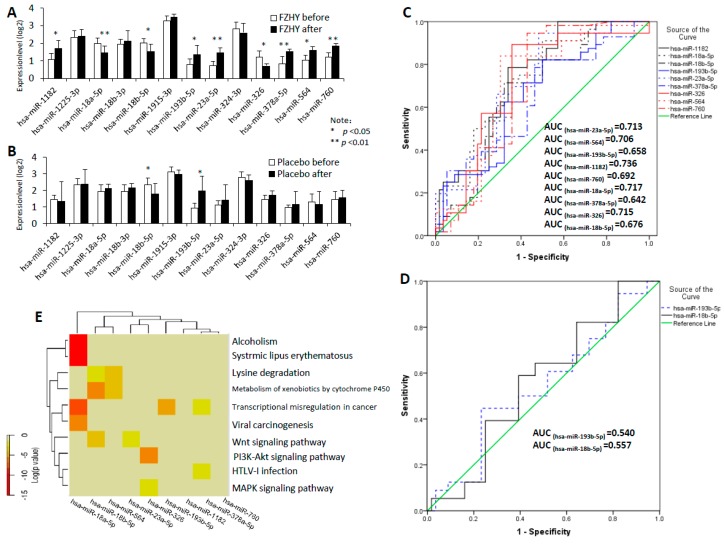
The potential kernel miRNAs were validated using the qPCR method. (**A**) Expression levels of 13 kernel miRNAs in the FZHY group. Illustrated *p*-values are based on pair-wise comparisons by the Mann–Whitney *U*-test. The result show that hsa-miR-1182, -18a-5p, -18b-5p, -193b-5p, -23a-5p, -326, -378a-5p, -564 and -760 (*p* < 0.05) have a statistically significant difference, while has-miR-1225-3p, -18b-3p, -1915-3p and -324-3p are insignificant between before and after trial; (**B**) Expression levels of 13 kernel miRNAs in the placebo group; hsa-miR-18b-5p and -193b-5p (*p* < 0.05) have significant differences between before and after trial. Illustrated *p*-values are based on pair-wise comparisons by the Mann–Whitney *U*-test; (**C**) Receiver operating characteristic (ROC) curves for hsa-miR-1182, -18a-5p, -18b-5p, -193b-5p, -23a-5p, -326, -378a-5p, -564 and -760 in the FZHY group; (**D**) ROC curves for hsa-miR-18b-5p and -193b-5p in the placebo group; (**E**) Hierarchical cluster and heat map of nine kernel miRNAs. The pathway analysis was performed using the miRPath program, and the significant pathways were determined when the *p*-value <0.001.

To explore whether the potential miRNA markers play important roles in FZHY treatment, we performed qPCR to measure the expression levels of 13 miRNAs from FZHY and placebo-treated HBC patients. At the transcript expression level, a statistical significance was noted for nine miRNAs before compared to after FZHY treatment, such as hsa-miR-1182, -18a-5p, -18b-5p, -193b-5p, -23a-5p, -326, -378a-5p, -564 and -760 (*p* < 0.05, Figure 4A). ROC analysis shows that these miRNAs are suitable to diagnose the clinical efficacy of FZHY (The area under the ROC-curve >0.60; Figure 4B). These results suggest that the miRNAs’ expressed levels are a benefit to the efficacy evaluation of FZHY formula in the HBC treatment process.

Furthermore, the miRPath (v2.0) analysis [25] shows that these nine miRNAs are mainly associated with alcoholism, systemic lupus erythematosus, lysine degradation, transcriptional misregulation in cancer, the metabolism of xenobiotics by cytochrome P450, human T-cell lymphotropic virus I (HTLV-I) infection, the Wnt signaling pathway, the PI3K-Akt signaling pathway and the MAPK signaling pathway (Figure 4E). This indicates that the FZHY can increase the expression levels of relative kernel miRNAs in HBC treatment progression and subsequently affect several biological processes.

As the placebo-control, we notice that the miRNAs hsa-miR-18b-5p and -193b-5p have significant differences in the placebo group (Figure 4C); however, their lower AUC (AUC_(hsa-miR-193b-5p)_ = 0.540 and AUC_(hsa-miR-18b-5p)_ = 0.557; Figure 4D) suggested that the placebo might have a temporary and limited effect for HBC treatment.

### 2.5. Identifying the Excellent miRNA Panel

**Figure 5:**
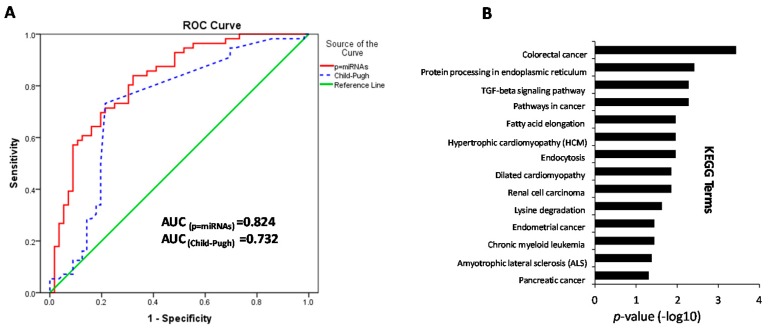
The KEGG terms of miRNA panel and the comparison of ROC between the miRNA panel and Child–Pugh. (**A**) ROC curves from the miRNA panel data and Child–Pugh data. ROC curve (*p* = miRNAs) generated using the miRNA expression data in the miRNA panel; the AUC was 0.824 (*p* = 0.000). The ROC curve of Child–Pugh was generated using the Child–Pugh score; the AUC was 0.732 (*p* = 0.000); (**B**) The miRNA combination of hsa-miR-18a-5p, -326, -1182 and -193b-5p associated with the KEGG pathway.

Using quantitative real-time polymerase chain reaction (qRT-PCR) data, a stepwise logistic regression model was designed to calculate the optimal combination from these nine miRNAs. Consequently, an excellent miRNA panel with hsa-miR-18a-5p, -326, -18a-5p, -1182 and -193b-5p was identified for the efficacy evaluation of FZHY. The logit model was (*p* = miRNAs) = 0.057 × hsa-miR-18a-5p − 0.036 × hsa-miR-326 + 0.049 × hsa-miR-1182 + 0.040 × hsa-miR-193b-5p + 1.266, which was used to construct the ROC-curve. The AUC for the test was 0.824 (95% CI: 0.747 to 0.901; Figure 5A). As a comparison, Child–Pugh scores of 180 samples (90 before and 90 after FZHY treatments) were used to predict the probability of FZHY clinical efficacy. The AUC for Child–Pugh was 0.732 (95% CI: 0.634 to 0.829; Figure 5A). This result suggested that multiple miRNAs can combined an excellent miRNA panel, and it obviously improves the diagnostic accuracy of drug evaluation; furthermore, it also implicates the differentially-expressed miRNA as an important biomarker for the efficacy evaluation of FZHY.

## 3. Discussion

As a Chinese herbal formula, FZHY was demonstrated to have a significant effect against hepatitis B-caused liver fibrosis; especially, it can be useful to improve HBC patient’s quality of life [8]. In this work, we designed a multicenter, double-blind, equally-randomized and placebo-controlled trial to evaluate the therapeutic efficacy of FZHY in HBC treatment progression. The results shown that the total Child-Pugh score has a statistically-significant difference before compared to after FZHY treatment, but it is inconspicuous in the placebo group. This suggested that the therapeutic efficacy of FZHY and placebo are different in trials, and FZHY is more advantageous for HBC treatment. Furthermore, 14 MIs were identified from 35 detected MIs before compared to after trials using the random variance model. Interestingly, some of the identified MIs might have random associations between before and after trials; in fact, they might provide useful ancillary functions to demonstrate the response to FZHY in the HBC treatment process. However, the conventional evaluation method is difficult with respect to distinguishing the tiny difference of the clinical efficacy between before and after FZHY treatment. Therefore, a timely and accurate diagnosis of the drug evaluation of FZHY is urgently needed.

In the FZHY group, 12 serum samples (six pairs before and after trials) were selected for miRNA profile detection, and 112 differentially-expressed miRNAs were identified, including 63 upregulated and 49 downregulated miRNAs. Generally, the exceptional stability of circulating miRNAs in serum is the basis of their value in clinical use [26]. To understand these miRNAs holistically, their regulated target genes were predicted by several programs, and then, enrichment analysis was conducted. The GO, KEGG and disease terms suggest that these miRNAs are mainly associated with signal-related pathways, such as the MAPK signaling pathway, the ErbB signaling pathway, the insulin signaling pathway, the mTOR signaling pathway, the TGF-β signaling pathway and the Wnt signaling pathway. In chronic liver disease, especially, TGF-β was considered as a central regulator, which contributed to all stages of disease progression from initial liver injury through inflammation and fibrosis to cirrhosis and hepatocellular carcinoma [27]. Many studies have identified the overexpression of TGF-β in various types of human cancer, which correlates with tumor progression, metastasis, angiogenesis and a poor prognostic outcome [28,29,30,31]. This implies that FZHY is able to significantly change the signal-related miRNAs expression levels, then mediates target genes and transforms the original progression of signal pathways in the HBC treatment process.

As a class of regulators, combinatorial regulation is an important feature in miRNA-regulated progression [32]. Such being the case, 13 kernel miRNAs were identified from the established miRNA-target network. Using quantitative RT-PCR, the expression levels of nine miRNAs have statistically-significant differences in the FZHY group (Figure 4A), and this suggested that they might play important roles in HBC treatment progression. Based on a stepwise logistic regression model, an excellent miRNA panel with hsa-miR-326, -18a-5p, -1182 and -193b-5p was identified from the validated miRNAs. The ROC curves demonstrated that this miRNA panel (AUC = 0.824; Figure 5A) is superior to each single miRNA (AUC = 0.658~0.736; Figure 4B), especially with accuracy higher than the Child–Pugh alone (AUC = 0.732; Figure 5A) in the diagnosis of FZHY curative estimation. This suggests that this miRNA panel can clearly improve the diagnostic accuracy of efficacy evaluation in FZHY treatment.

Furthermore, the KEGG terms show that this miRNA panel mainly tends to be expressed across multiple pathways, such as colorectal cancer (*p* = 0.0003), protein processing in endoplasmic reticulum (*p* = 0.0004), the TGF-β signaling pathway (*p* = 0.005) and pathways in cancer (*p* = 0.005) (Figure 5B). In the miRNA panel, the studies reported that hsa-miR-18a-5p can significantly reduce the hazard of dying from colorectal cancer [33]; hsa-miR-1182 can suppress the TERT gene expression level and might act as an effective target for gastric cancer treatment [34]. In human lymphomas, hsa-miR-193b clearly contributes to enhancing the expression of the *SMO* gene and subsequently activating the GLI/Hh signaling [35]. Furthermore, hsa-miR-326 was considered as an important regulator and to be correlated with several human cancers, such as glioma [36], esophageal cancer [37], head and neck cancer [38] and hepatocellular carcinoma [39]. Importantly, hsa-miR-326 is a central mediator in the pathogenesis of pulmonary fibrosis, which acts by affecting multiple components of the TGF-β signaling pathway [40]. These studies indicated this miRNA panel might act as potential circulating markers for the efficacy evaluation of FZHY in the HBC treatment process.

## 4. Materials and Methods

### 4.1. Overview of the Framework

**Figure 6:**
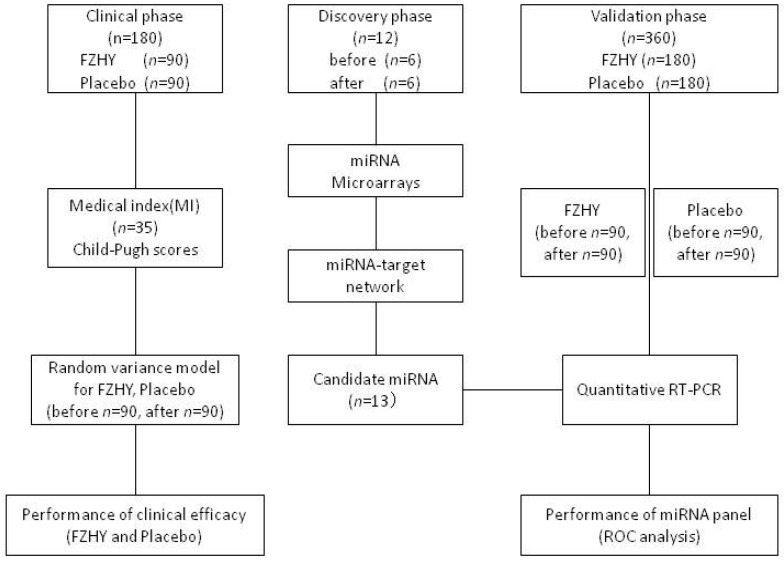
Overview of the framework. FZHY: Fuzheng-Huayu (Chinese herbal formula); MI: medical index; RT-PCR: reverse transcriptase polymerase chain reaction; ROC: receiver operating characteristic.

This study focuses on the identification of potential miRNA markers in HBC treatment progression with FZHY formula. At first, the multicenter, double-blind, equally-randomized and placebo-controlled trial was used to analyze the clinical efficacy of FZHY in HBC treatment. Morning fasting venous blood samples from 180 patients (90 cases with FZHY treatment and 90 cases with placebo treatment) were collected individually, which were used for medical indexes’ (MI, *n* = 35) detection. After 6 months of treatment, the same works were performed in these 180 cases. Secondly, 12 serum samples (6 pairs of before and after FZHY treatment) were used for miRNA microarray detection. The differentially-expressed miRNAs between before and after FZHY treatment were identified, and the miRNA-target network was constructed to identify the kernel miRNA markers. Lastly, 360 serum samples (FZHY before *n* = 90 and after *n* = 90; placebo before *n* = 90 and after *n* = 90) were used to validate the probability of potential miRNA markers in FZHY treatment of HBC patients (Figure 6). The serum samples were stored at −80 °C.

The clinical specimens from HBC patients and healthy donors were collected from Shanghai Shuguang Hospital, and all subjects gave informed consent for this trial. The diagnostic criteria of Western medicine for HBC followed the guidelines defined by the Chinese Society of Hepatology and Chinese Society of Infectious Diseases in 2005 [41]. This research project was carried out in accordance with the approved guidelines (identification code: 06DZ19728) of the Declaration of Helsinki and the principles of good clinical practice (Shanghai, China) and were approved by the local ethics committee of Shanghai University of Traditional Chinese Medicine (Shanghai, China).

### 4.2. Clinical Specimens

In the trial, participants have been randomly given FZHY and placebo (2% FZHY), and then, 35 medical indexes (MIs) were detected from 90 FZHY- and 90 placebo-treated HBC patients, respectively. After 6 months of treatment, the same works were performed in these 180 cases. The Child–Pugh total score was calculated from ascites (Asc), bilirubin (BIL), albumin (ALB), prothrombin time (PT) and hepatic encephalopathy (HE). The clinical data of subjects are presented in Table 2.

### 4.3. Clinical Data Analysis

Using the R package, a random variance model was designed to test the *p*-value of medical indexes. In the model, *i* represent the distribution of each medical index expression level between before and after treatment.

(1) $$t=\frac{{\bar{x}}_{i,1}-{\bar{x}}_{i,2}}{{\tilde{\sigma }}_{i}\sqrt{\frac{1}{n_{1}}+\frac{1}{n_{2}}}}$$

(2) σ˜i2=(n1+n2−2)σ^i2+2a(1ab)(n1+n2−2)+2a

where the numerator is the difference in the means of the log value *i* in the two classes (FZHY and placebo), *n*_1_ and *n*_2_ are the number of samples in the two classes, ${\tilde{\sigma }}_{i}^{2}$ is a weighted average of the usual medical index-specific variance and the average variance for all indexes and ${\hat{\sigma }}_{i}$ denotes the square root of the usual within-class variance for index *i*. The 1/*ab* is an estimate of the expected variance for the inverse γ model, and 2*a* represents the average variance for all indexes.

### 4.4. miRNA Profiles Detection

The Child–Pugh score was used to identify the selected samples from FZHY-treated HBC patients. If the Child–Pugh score has a statistically-significant decrease after the trials, this patient was considered to respond to FZHY and to act as a candidate subject. Based on this, 6 pairs of serum samples (6 samples before trial and 6 samples after trial) were selected for miRNA profile detection. The miRNA profiles were generated using Agilent Human miRNA microarray V3 (Agilent Technologies Inc., Santa Clara, CA, USA). All raw data were transformed to log2, and each expression was normalized by zero mean and unit sample variance. Using the SAM (significance analysis of microarrays) method of the R package, the differentially-expressed (DE) miRNAs were identified before compare to after FZHY treatment, where the fold-change >1.5 and *p* < 0.001 were considered to be significant.

### 4.5. miRNA Target Genes Prediction

The miRNA target genes were predicted using three databases, TarBase (v7.0) [42], miRecords [43] and miRTarBase [44], which contained the largest collection of manually-curated experimental data. Furthermore, miRanda, miRDB, miRWalk and RNAhybrid programs were used to predict the non-experimental targets, where the *p* < 0.001 was considered to be significant. The predicted target genes were analyzed using DAVID online [20,21]; significance analysis was defined as the *p*-value adjusted by the false discovery rate (FDR), and gene sets containing less than 5 genes overlapping were removed. In this study, Gene Ontology (GO), pathway and the disease term with an FDR-adjusted *p*-value of less than 0.05 were retained.

### 4.6. miRNA-Target Network Construction

The differentially-expressed (DE) miRNAs and predicted targets were combined to construct the miRNA-target network. miRNAs were weighted by the fold change (|log2|); target genes were weighted based on the distribution of degrees. Subsequently, all nodes were ranked according to their weights, and the similarity was tested; then, the obtained deregulated nodes were used to remap the network. In the network, the node represents the miRNA or target; the edge represents the connection strength. The topological profiles of the network were calculated by the ClusterONE algorithm [24]; consecutive clusters were generated, which were defined as a *p*-value <0.0001, node size >5, quality >0.60 and density >0.05. The determined miRNA clusters were considered as kernel miRNAs and play important roles in HBC treatment progression.

### 4.7. Quantification of Potential Marker miRNA

The qRT-PCR was used to identify the kernel miRNAs in 360 serum samples (FZHY before *n* = 90 and after *n* = 90; placebo before *n* = 90 and after *n* = 90). The quantification of miRNA was performed with SYBR Green PCR Master Mixture (TOYOBO Biotech Co., Ltd., Osaka, Japan) according to the manufacturer’s instructions using a Rotor-Gene 6000 Real-time PCR machine (Corbett Life Science, Sydney, Australia). The specificity of each PCR product was validated by the melting curve at the end of PCR cycles. All miRNAs were validated in triplicate; the *C*_t_ was considered as the number of cycles required and for the fluorescent signal to reach the threshold. The levels of miRNAs were calculated using 2Δ*C*_t_, where Δ*C*_t_ = *C*_t_ of the internal reference −*C*_t_ of the target miRNA. The differences in miRNAs’ expression levels between groups were compared using the Student’s *t*-test, and a *p*-value <0.05 was considered to be a statistically-significant difference.

### 4.8. Experimental Data Analysis

Using qRT-PCR data, a stepwise logistic regression model was used to screen diagnostic miRNA markers. The predicted probability of being diagnosed with FZHY therapeutic efficacy was used as a surrogate marker to construct a receiver operating characteristic (ROC) curve. The area under the ROC-curve (AUC) was used as an accuracy index for evaluating the diagnostic performance of the selected miRNA panel. All of the tests were two-tailed, and *p* < 0.05 was considered statistically significant.

## 5. Conclusions

In conclusion, we demonstrated the transcriptional profiles, differentially-expressed miRNAs and their functional properties to evaluate the therapeutic efficacy of FZHY in HBC treatment progression. This analysis suggested that several interesting miRNAs, including hsa-miR-18a-5p, -326, -1182 and -193b-5p, are potential biomarkers for FZHY efficacy evaluation and may be helpful in the development of noninvasive drug evaluating methods in the future.

## Figures and Tables

**Table 1:** The distribution of potential miRNA markers from the network.

Cluster	Kernel miRNA	Nodes	Density	Quality	*p*-Value	Status ^a^
1	hsa-miR-1225-3p	36	0.055	0.625	0	up
2	hsa-miR-18a-5p	67	0.057	0.626	0	down
	hsa-miR-18b-5p					down
3	hsa-miR-378a-5p	40	0.051	0.6396	0	up
4	hsa-miR-1915-3p	29	0.068	0.667	1.41 × 10^−8^	up
5	hsa-miR-760	22	0.090	0.750	5.40 × 10^−8^	up
6	hsa-miR-1182	24	0.0833	0.697	4.08 × 10^−7^	up
7	hsa-miR-326	23	0.087	0.629	9.07 × 10^−6^	down
8	hsa-miR-23a-5p	18	0.111	0.680	2.87 × 10^−5^	up
9	hsa-miR-324-3p	13	0.154	0.750	7.74 × 10^−5^	up
10	hsa-miR-564	8	0.250	0.875	4.84 × 10^−4^	up
11	hsa-miR-18b-3p	18	0.111	0.630	5.68 × 10^−4^	up
12	hsa-miR-193b-5p	14	0.143	0.6840	9.15 × 10^−4^	up

^a^ miRNA expression status in the microarray after Fuzheng-Huayu (FZHY) treatment.

**Table 2:** Clinical data of subjects ^a^.

Characteristics	FZHY Untreated (Mean ± SD)	FZHY Treated (Mean ± SD)	Placebo Untreated (Mean ± SD)	Placebo Treated (Mean ± SD)
Age (years)	50.6 ± 8.5		50.3 ± 8.3	
Gender				
Male (*n*)	60		64	
Female (*n*)	30		26	
Total	90		90	
HBV History (years)	14.1 ± 10.3		14.2 ± 10.7	
ALT (U/L)	49.93 ± 44.40	39.76 ± 16.17	52.09 ± 51.09	45.48 ± 31.51
AST (U/L)	58.66 ± 44.81	54.92 ±39.07	61.46 ± 47.14	57.51 ± 40.59
GGT (U/L)	57.61 ± 55.77	64.88 ± 75.24	59.22± 66.14	66.36 ± 81.41

^a^ SD, standard deviation.

## Abbreviations

FZHY	Fuzheng-Huayu
DE	differentially expressed
miRNA	microRNA

## References

[1] Hsu A., Lai C.L., Yuen M.F. (2011). Update on the risk of hepatocellular carcinoma in chronic hepatitis b virus infection. Curr. Hepat. Rep..

[2] De Jongh F.E., Janssen H.L., de Man R.A., Hop W.C., Schalm S.W., van Blankenstein M. (1992). Survival and prognostic indicators in hepatitis B surface antigen-positive cirrhosis of the liver. Gastroenterology.

[3] Tan Y.J. (2011). Hepatitis B virus infection and the risk of hepatocellular carcinoma. World J. Gastroenterol..

[4] Dong S., Chen Q.L., Su S.B. (2015). Curative effects of fuzheng huayu on liver fibrosis and cirrhosis: A meta-analysis. Evid. Based Complement. Altern. Med..

[5] Yang T., Shen D.P., Wang Q.L., Tao Y.Y., Liu C.H. (2013). Investigation of the absorbed and metabolized components of danshen from Fuzheng Huayu recipe and study on the anti-hepatic fibrosis effects of these components. J. Ethnopharmacol..

[6] Liu C., Hu Y., Xu L., Liu C., Liu P. (2009). Effect of Fuzheng Huayu formula and its actions against liver fibrosis. Chin. Med..

[7] Liu P., Hu Y.Y., Liu C., Xu L.M., Liu C.H., Sun K.W., Hu D.C., Yin Y.K., Zhou X.Q., Wan M.B. (2005). Multicenter clinical study on Fuzhenghuayu capsule against liver fibrosis due to chronic hepatitis B. World J. Gastroenterol..

[8] Song Y.N., Sun J.J., Lu Y.Y., Xu L.M., Gao Y.Q., Zhang W., Wang X.S., Xue D.Y., Zheng Q.S., Su S.B. (2013). Therapeutic efficacy of Fuzheng-Huayu tablet based traditional chinese medicine syndrome differentiation on hepatitis-B-caused cirrhosis: A multicenter double-blind randomized controlled trail. Evid. Based Complement. Altern. Med..

[9] Sun S., Dai J., Wang W., Cao H., Fang J., Hu Y.Y., Su S., Zhang Y. (2012). Metabonomic evaluation of zheng differentiation and treatment by Fuzhenghuayu tablet in hepatitis-B-caused cirrhosis. Evid. Based Complement. Altern. Med..

[10] Tao Y.Y., Yan X.C., Zhou T., Shen L., Liu Z.L., Liu C.H. (2014). Fuzheng Huayu recipe alleviates hepatic fibrosis via inhibiting TNF-α induced hepatocyte apoptosis. BMC Complement. Altern. Med..

[11] Wang Q.L., Yuan J.L., Tao Y.Y., Zhang Y., Liu P., Liu C.H. (2010). Fuzheng Huayu recipe and vitamin E reverse renal interstitial fibrosis through counteracting TGF-β1-induced epithelial-to-mesenchymal transition. J. Ethnopharmacol..

[12] Wang Q.L., Tao Y.Y., Shen L., Cui H.Y., Liu C.H. (2012). Chinese herbal medicine Fuzheng Huayu recipe inhibits liver fibrosis by mediating the transforming growth factor-β1/smads signaling pathway. Zhong Xi Yi Jie He Xue Bao.

[13] Wang R.Q., Mi H.M., Li H., Zhao S.X., Jia Y.H., Nan Y.M. (2015). Modulation of IKKΒ/NF-κB and TGF-β1/SMAD via Fuzheng Huayu recipe involves in prevention of nutritional steatohepatitis and fibrosis in mice. Iran. J. Basic Med. Sci..

[14] Lok A.S., Ghany M.G., Goodman Z.D., Wright E.C., Everson G.T., Sterling R.K., Everhart J.E., Lindsay K.L., Bonkovsky H.L., di Bisceglie A.M. (2005). Predicting cirrhosis in patients with hepatitis C based on standard laboratory tests: Results of the halt-C cohort. Hepatology.

[15] Koda M., Matunaga Y., Kawakami M., Kishimoto Y., Suou T., Murawaki Y. (2007). Fibroindex, a practical index for predicting significant fibrosis in patients with chronic hepatitis C. Hepatology.

[16] Castera L. (2012). Noninvasive methods to assess liver disease in patients with hepatitis B or C. Gastroenterology.

[17] Hou J., Lin L., Zhou W., Wang Z., Ding G., Dong Q., Qin L., Wu X., Zheng Y., Yang Y. (2011). Identification of mirnomes in human liver and hepatocellular carcinoma reveals miR-199a/b-3p as therapeutic target for hepatocellular carcinoma. Cancer Cell.

[18] Forman J.J., Legesse-Miller A., Coller H.A. (2008). A search for conserved sequences in coding regions reveals that the Let-7 microRNA targets dicer within its coding sequence. Proc. Natl. Acad. Sci. USA.

[19] Lytle J.R., Yario T.A., Steitz J.A. (2007). Target mRNAs are repressed as efficiently by microRNA-binding sites in the 5′ UTR as in the 3′ UTR. Proc. Natl. Acad. Sci. USA.

[20] Huang da W., Sherman B.T., Lempicki R.A. (2009). Systematic and integrative analysis of large gene lists using david bioinformatics resources. Nat. Protoc..

[21] Huang da W., Sherman B.T., Lempicki R.A. (2009). Bioinformatics enrichment tools: Paths toward the comprehensive functional analysis of large gene lists. Nucleic Acids Res..

[22] Guo Y., Feng Y., Trivedi N.S., Huang S. (2011). Medusa structure of the gene regulatory network: Dominance of transcription factors in cancer subtype classification. Exp. Biol. Med. Maywood.

[23] Chen Q.L., Lu Y.Y., Zhang G.B., Song Y.N., Zhou Q.M., Zhang H., Zhang W., Tang X.S., Su S.B. (2013). Characteristic analysis from excessive to deficient syndromes in hepatocarcinoma underlying miRNA array data. Evid. Based Complement. Altern. Med..

[24] Nepusz T., Yu H., Paccanaro A. (2012). Detecting overlapping protein complexes in protein–protein interaction networks. Nat. Methods.

[25] Vlachos I.S., Kostoulas N., Vergoulis T., Georgakilas G., Reczko M., Maragkakis M., Paraskevopoulou M.D., Prionidis K., Dalamagas T., Hatzigeorgiou A.G. (2012). DIANA miRpath v.2.0: Investigating the combinatorial effect of microRNAs in pathways. Nucleic Acids Res..

[26] Schrauder M.G., Strick R., Schulz-Wendtland R., Strissel P.L., Kahmann L., Loehberg C.R., Lux M.P., Jud S.M., Hartmann A., Hein A. (2012). Circulating micro-RNAs as potential blood-based markers for early stage breast cancer detection. PLoS ONE.

[27] Dooley S., ten Dijke P. (2012). TGF-β in progression of liver disease. Cell Tissue Res..

[28] Zhang L., Zhou F., Garcia de Vinuesa A., de Kruijf E.M., Mesker W.E., Hui L., Drabsch Y., Li Y., Bauer A., Rousseau A. (2013). TRAF4 promotes TGF-β receptor signaling and drives breast cancer metastasis. Mol. Cell.

[29] Saito A., Suzuki H.I., Horie M., Ohshima M., Morishita Y., Abiko Y., Nagase T. (2013). An integrated expression profiling reveals target genes of TGF-β and TNF-α possibly mediated by microRNAs in lung cancer cells. PLoS ONE.

[30] Lv S., Qin J., Yi R., Coreman M., Shi R., Kang H., Yao C. (2013). Crkl efficiently mediates cell proliferation, migration, and invasion induced by TGF-β pathway in glioblastoma. J. Mol. Neurosci. MN.

[31] Mu X., Lin S., Yang J., Chen C., Chen Y., Herzig M.C., Washburn K., Halff G.A., Walter C.A., Sun B. (2013). TGF-β signaling is often attenuated during hepatotumorigenesis, but is retained for the malignancy of hepatocellular carcinoma cells. PLoS ONE.

[32] Chen Q.-L., Lu Y.-Y., Zhang G.-B., Song Y.-N., Zhou Q.-M., Zhang H., Zhang W., Su S.-B. (2013). Progression from excessive to deficient syndromes in chronic hepatitis B: A dynamical network analysis of miRNA array data. Evid. Based Complement. Altern. Med..

[33] Slattery M.L., Herrick J.S., Mullany L.E., Valeri N., Stevens J., Caan B.J., Samowitz W., Wolff R.K. (2015). An evaluation and replication of miRNAs with disease stage and colorectal cancer-specific mortality. Int. J. Cancer.

[34] Zhang D., Xiao Y.F., Zhang J.W., Xie R., Hu C.J., Tang B., Wang S.M., Wu Y.Y., Hao N.B., Yang S.M. (2015). MiR-1182 attenuates gastric cancer proliferation and metastasis by targeting the open reading frame of htert. Cancer Lett..

[35] Gonzalez-Gugel E., Villa-Morales M., Santos J., Bueno M.J., Malumbres M., Rodriguez-Pinilla S.M., Piris M.A., Fernandez-Piqueras J. (2013). Down-regulation of specific miRNAs enhances the expression of the gene smoothened and contributes to T-cell lymphoblastic lymphoma development. Carcinogenesis.

[36] Du W., Liu X., Chen L., Dou Z., Lei X., Chang L., Cai J., Cui Y., Yang D., Sun Y. (2015). Targeting the SMO oncogene by miR-326 inhibits glioma biological behaviors and stemness. Neuro Oncol..

[37] Hong C.C., Chen P.S., Chiou J., Chiu C.F., Yang C.Y., Hsiao M., Chang Y.W., Yu Y.H., Hung M.C., Hsu N.W. (2014). MiR326 maturation is crucial for VEGF-C-driven cortactin expression and esophageal cancer progression. Cancer Res..

[38] Lindenbergh-van der Plas M., Martens-de Kemp S.R., de Maaker M., van Wieringen W.N., Ylstra B., Agami R., Cerisoli F., Leemans C.R., Braakhuis B.J., Brakenhoff R.H. (2013). Identification of lethal microRNAs specific for head and neck cancer. Clin. Cancer Res..

[39] Zhang J., Chong C.C., Chen G.G., Lai P.B. (2015). A seven-microRNA expression signature predicts survival in hepatocellular carcinoma. PLoS ONE.

[40] Das S., Kumar M., Negi V., Pattnaik B., Prakash Y.S., Agrawal A., Ghosh B. (2014). MicroRNA-326 regulates profibrotic functions of transforming growth factor-β in pulmonary fibrosis. Am. J. Respir. Cell Mol. Biol..

[41] Hepatology C.S.O., Association C.M., Diseases C.S.O.I., Association C.M. (2007). Guideline on prevention and treatment of chronic hepatitis B in China (2005). Chin. Med. J..

[42] Vlachos I.S., Paraskevopoulou M.D., Karagkouni D., Georgakilas G., Vergoulis T., Kanellos I., Anastasopoulos I.L., Maniou S., Karathanou K., Kalfakakou D. (2015). DIANA-TarBase v7.0: Indexing more than half a million experimentally supported miRNA:mRNA interactions. Nucleic Acids Res..

[43] Xiao F., Zuo Z., Cai G., Kang S., Gao X., Li T. (2009). Mirecords: An integrated resource for microRNA-target interactions. Nucleic Acids Res..

[44] Hsu S.D., Lin F.M., Wu W.Y., Liang C., Huang W.C., Chan W.L., Tsai W.T., Chen G.Z., Lee C.J., Chiu C.M. (2011). Mirtarbase: A database curates experimentally validated microRNA-target interactions. Nucleic Acids Res..

